# A Visual Method for the Safe Administration of Multiple Local Anesthetics

**DOI:** 10.1097/GOX.0000000000002294

**Published:** 2019-06-19

**Authors:** Sarah M. Budney, Travis C. Holcombe

**Affiliations:** From the Department of Surgery, Creighton University School of Medicine, Phoenix, Ariz.

The role of local anesthetic (LA) as an alternative or additive to general anesthesia has revolutionized the methods of analgesia used during procedures.^1^ Although there are studies to guide the dosing of individual LAs, there have not been studies to guide dosing when administering multiple LAs simultaneously. However, it is the practice of many surgeons to mix or use multiple LAs to have the benefits of each agent.^1^ Current recommendations are to not exceed the combined maximum dose.^2^ Meaning, if you have given 50% of the maximum dose of one agent, you should not administer more than 50% of the maximum dose of the other agent. However, these calculations can be difficult to complete intraoperatively as exact doses of each LA are not always predetermined before administration.

Local anesthetics work by inhibiting voltage-dependent sodium channels, which reduces the influx of sodium ions and thus conduction of neuronal impulses.^2^ Their mechanism makes them particularly dangerous if injected into a vessel or given in excessive doses as they work on all excitable membranes. In severe cases, toxicity may result in a life-threatening condition known as local anesthetic systemic toxicity in which the patient has cardiovascular and neurological collapse. Hence, for clinicians who choose to use more than one LA at a time, it is imperative that they do not exceed the recommended maximum dose. Here, we discuss a plastic surgeon’s method for determining dosing combinations when using more than one local anesthetic, which allows for intraoperative adjustments.

**Fig. 1.**
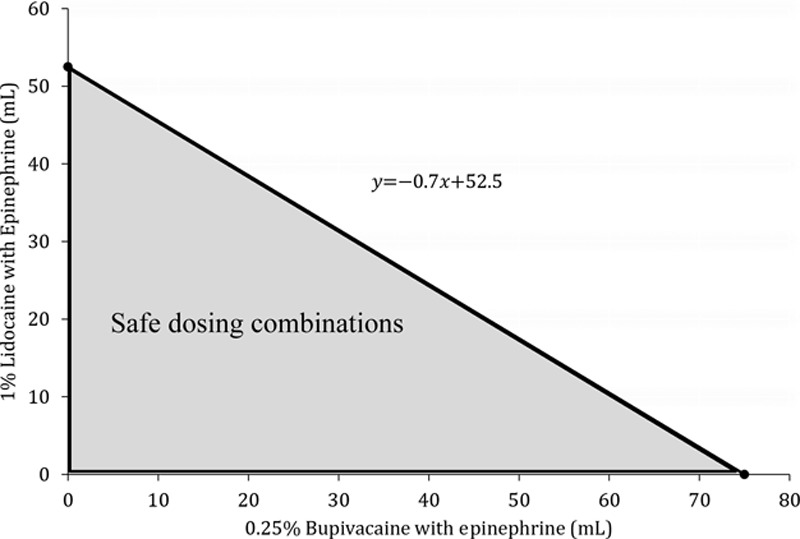
Example graph of safe dosing combinations for a 75-kg male receiving 1% lidocaine with epinephrine and 0.25% bupivacaine with epinephrine.

Before the surgery, the maximum dose of each agent is calculated by volume. Then a linear equation is generated such that the *y*- and *x*-intercept are the maximum doses of each agent. For example, take a 75-kg male who is undergoing a procedure in which both 1% lidocaine with epinephrine and 0.25% bupivacaine with epinephrine will be used. Then, the maximum dose of each agent is 52.5 and 75 mL, respectively. If we assign the dose of 1% lidocaine with epinephrine in mL to the *y* axis and the dose of 0.25% bupivacaine with epinephrine in mL to the *x* axis, then the equation of the line will be y=-0.7x+52.5 (Fig. 1). The line itself represents all possible combinations of the maximum combined dose and any combination below the line is considered to be safe. Using this line, one can easily visualize how much of each agent they can administer safely.

Although the use of multiple LAs in a single case is largely discouraged due to the lack of evidence on safe dosing, it is still a technique commonly employed by surgeons and other medical professionals.^2^ It may be difficult to adhere to dosing recommendation intraoperatively because of its mathematical nature. With the use of a simple graph, however, clinicians can easily alter doses while ensuring they are in a safe range.
